# Umbilical port-site endometriosis: a case presentation and narrative review of the literature

**DOI:** 10.1515/med-2026-1411

**Published:** 2026-04-09

**Authors:** Pawel Sadlecki, Katarzyna Dejewska, Malgorzata Walentowicz-Sadlecka

**Affiliations:** Faculty of Medicine, University of Science and Technology, Bydgoszcz, Poland; Department of Obstetrics, Gynecology and Gynecologic Oncology, Regional Polyclinical Hospital, Grudziadz, Poland

**Keywords:** abdominal wall endometriosis, umbilical port-site endometriosis, scar endometriosis, laparoscopy, trocar site

## Abstract

**Introduction:**

Umbilical port-site endometriosis is a rare form of abdominal wall endometriosis (AWE), especially following non-gynecologic surgeries. Diagnosing AWE is challenging because it mimics other abdominal wall masses.

**Content:**

We describe a 46-year-old woman with cyclic umbilical pain and a mass at a previous trocar site, 17 years after laparoscopic cholecystectomy. Her history included two earlier cesarean sections, both recognized risk factors for iatrogenic AWE. Imaging demonstrated a vascularized lesion. Surgical excision confirmed endometriosis. A narrative review of the literature (2000–2025) was conducted using PubMed, Scopus, and Google Scholar. Broad inclusion criteria were applied; SANRA principles guided quality appraisal. A basic quantitative summary of reported cases was included.

**Summary:**

Twenty-seven relevant publications were included. Port-site AWE remains rare, and standardized diagnostic pathways and follow-up protocols are lacking. Most cases were managed with wide local excision with negative margins. Recurrence data are sparse due to short or absent follow-up in many reports.

**Outlook:**

Clinicians – particularly general surgeons – should consider AWE in patients with painful masses at trocar or scar sites. Although the temporal association with cholecystectomy raises suspicion, prior cesarean sections provide a more plausible etiological source in this case. Long-term follow-up and international registries are needed to better characterize outcomes of port-site endometriosis.

## Introduction

Endometriosis, classically defined as the presence of endometrial glands and stroma in extrauterine locations, affects 6–10 % of women of reproductive age [1]. The most common symptoms of endometriosis include dysmenorrhea, dyspareunia, chronic pelvic pain, and infertility [2]. Endometriosis can be classified as intrapelvic or extrapelvic. Intrapelvic endometriosis includes primarily the pelvic peritoneum, ovaries, and rectovaginal septum. Extrapelvic sites have been reported in abdominal wall, extremities, lungs, and brain [3]. While most lesions occur within the pelvis, extrapelvic endometriosis also appears in the abdominal wall, typically associated with prior surgical incisions. Abdominal wall endometriosis (AWE), particularly when involving the rectus abdominis at laparoscopic trocar or incision sites, is exceptionally rare – accounting for just 0.03–2 % of extrapelvic cases [4]. It typically arises after gynecological or obstetric surgeries such as laparotomy or laparoscopy, where ectopic endometrial tissue becomes implanted in subcutaneous fat and muscle layers, presenting as cyclic pain or palpable nodules near surgical scars [5]. The diagnosis of AWE is often difficult, as it can mimic a variety of benign and malignant abdominal wall masses. Due to its deep location and nonspecific presentation, diagnosis often requires imaging (e.g., ultrasound, CT, MRI) followed by confirmatory histopathology; surgical excision with clear margins remains the definitive treatment [5].

The present study describes a case of umbilical port-site endometriosis with a 17-year latency and provides a narrative review of the literature, emphasizing diagnostic challenges, differential diagnosis, and optimal surgical management.

## Case presentation

**Figure 1:**
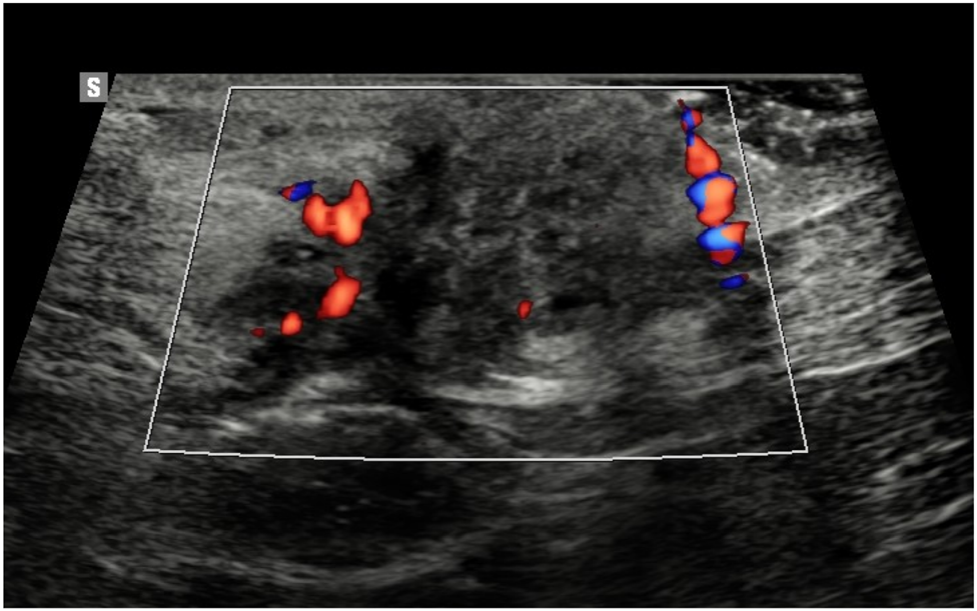
Ultrasound image of the umbilical mass demonstrating a solid, heterogeneous lesion with peripheral vascularization on Doppler, consistent with characteristics of abdominal wall endometriosis.

**Figure 2:**
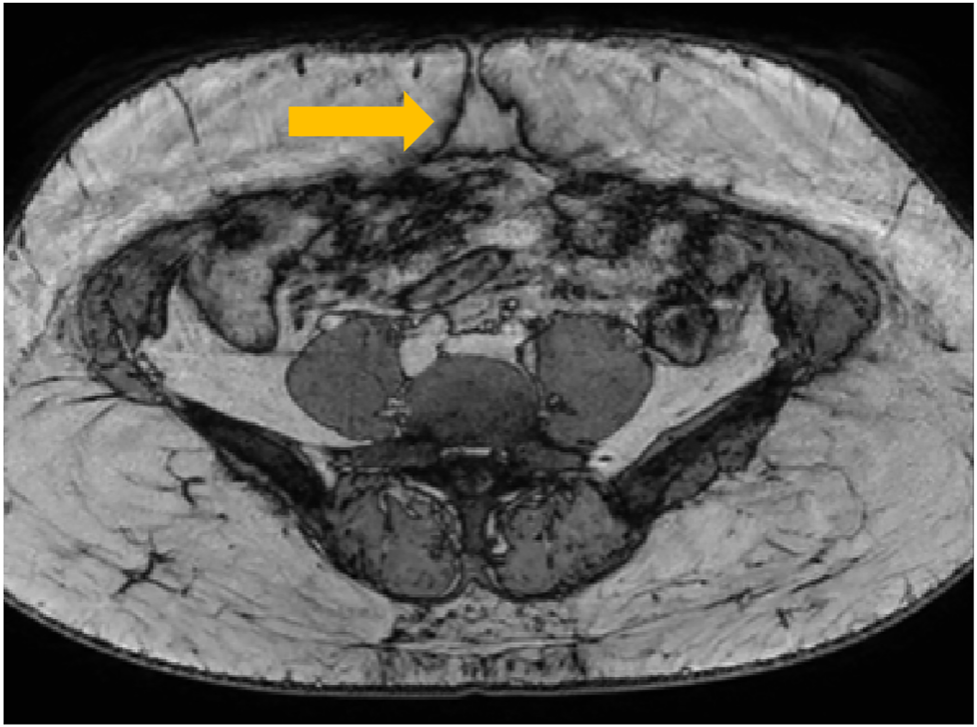
MRI of the abdominal wall showing a contrast-enhancing mass localized within the rectus muscle (yellow arrow), without diffusion restriction, aiding in preoperative characterization and surgical planning.

**Figure 3:**
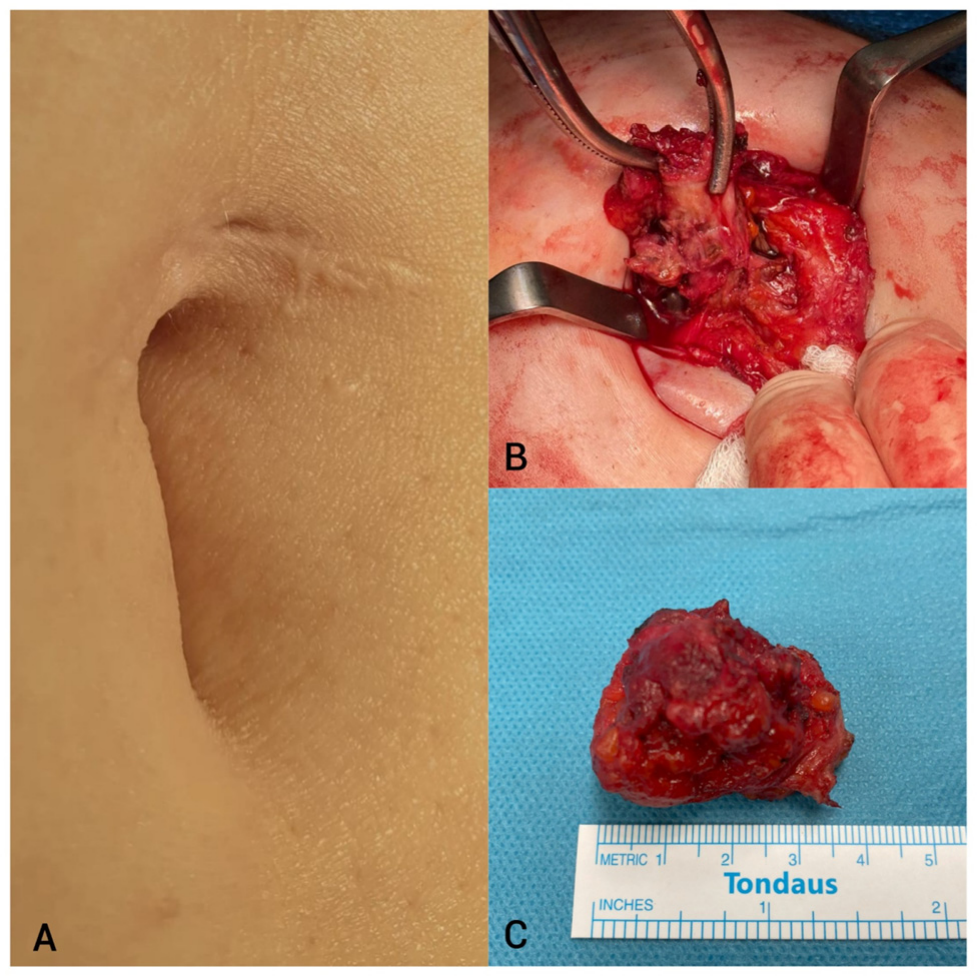
Umbilical port-site endometriosis. (A) The lesion was not macroscopically visible; however, it was clearly palpable beneath the scar from the optical trocar in the umbilicus. (B) An irregular fibro-fatty mass was excised en bloc with a surrounding tissue margin. (C) Tumour excised from the muscular layer of the abdominal wall.

A 46-year-old woman presented with a slowly enlarging nodule beneath her umbilical trocar-site scar from a previous laparoscopic cholecystectomy, reporting cyclic peri-menstrual pain. The patient reported having undergone two cesarean sections (in 2003 and 2007) as well as a laparoscopic cholecystectomy in 2008. She experienced pain for approximately two years, with symptoms occurring predominantly during menstruation and persisting up to 12 days afterward. Laboratory tests revealed no significant deviations from normal. Imaging studies (ultrasound and MRI) identified a vascularized mass measuring 21 × 32 × 36 mm in the umbilical region. Color Doppler ultrasound demonstrated peripheral vascular flow (Figure 1), and MRI showed contrast enhancement without diffusion restriction of the mass located within the muscular layer of the abdominal wall (Figure 2). In a prior biopsy of the lesion, the presence of malignancy was excluded. The patient was scheduled for surgical excision of the lesion via the umbilical skin scar. An irregular fibro-fatty mass measuring approximately 32 × 24 × 36 mm was excised en bloc with a surrounding tissue margin (Figure 3). On gross examination, the specimen displayed a yellow-brown cut surface with focal hemorrhagic areas. Histological analysis confirmed the presence of endometrial glands and stroma, consistent with a diagnosis of endometriosis. The patient’s postoperative course was uneventful, and she was discharged home on the second postoperative day. As adjuvant therapy, she was prescribed dienogest at a dose of 2 mg once daily.

## Materials and methods

We conducted a narrative literature review guided by established best practices. The SANRA (Scale for the Assessment of Narrative Review Articles) checklist was applied to assess the methodological quality of the selected narrative review articles. After clearly defining our objectives (diagnosis, differential diagnosis, and treatment of port site endometriosis), we searched PubMed, Scopus, and Google Scholar (January 2000–January 2025) using the following keywords and Boolean combinations: port-site endometriosis, umbilical trocar site endometriosis, abdominal wall endometriosis, scar endometriosis, laparoscopy complications. Reference lists of eligible articles were also screened.

**Figure 4:**
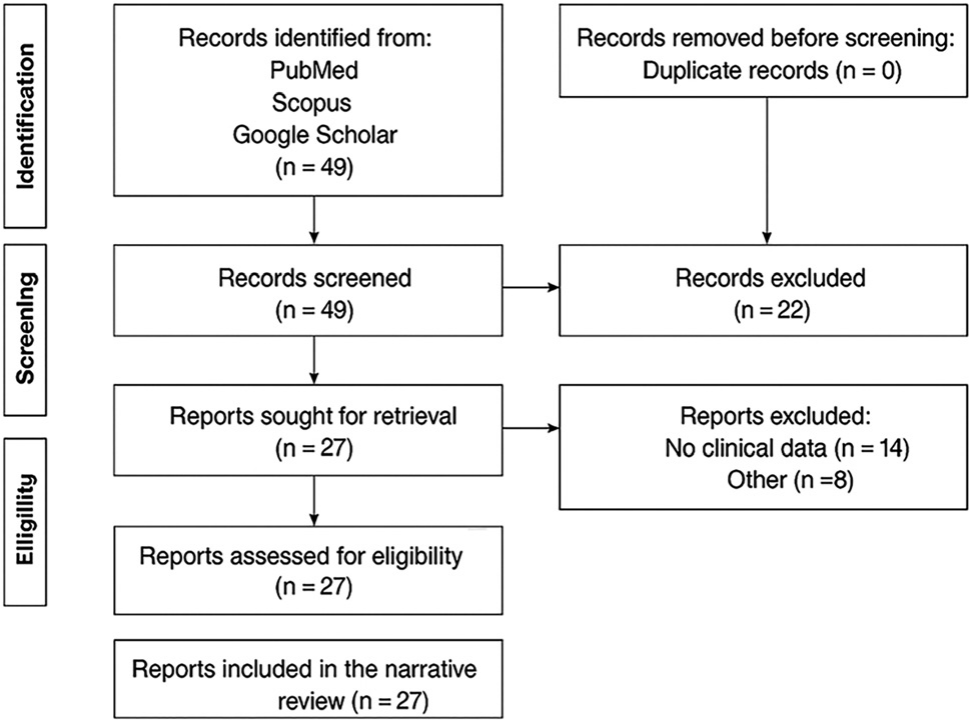
The flow diagram of the literature search and study selection.

Articles were selected based on relevance and content depth, using the inclusion and exclusion criteria listed below. Inclusion criteria were: case reports, case series, observational studies, reviews, and expert recommendations on AWE or port-site endometriosis, sufficient clinical or imaging information. Exclusion criteria were: experimental molecular studies without clinical data, studies not involving abdominal wall or trocar-site disease. The search identified 49 unique articles; 27 met inclusion criteria. Two authors independently screened abstracts and full texts. The flow diagram of the literature search and study selection process is presented in Figure 4. We extracted and thematically synthesized information – focusing on diagnostic modalities, surgical approaches – while critically appraising heterogeneity in methods and findings. The results were limited to the English language. This flexible, interpretive approach allowed for a comprehensive overview of current knowledge, identification of research gaps, and contextual understanding of evolving surgical practices.

### Ethical approval

This project was conducted in accordance with the Declaration of Helsinki and approved by the Bioethics Committee of the Kujawsko-Pomorskie Regional Chamber of Physicians in Toruń (approval no. 20/KB/2023).

### Informed consent

Written informed consent was obtained from the patient for publication of clinical details and images.

## Discussion

AWE typically presents as a nodule along a surgical scar. With the widespread adoption of laparoscopic surgery, reports of endometriosis occurring at trocar incision sites have been rare. Most experts believe that AWE results from the implantation of endometrial cells during surgical procedures [6].

The exact pathogenesis of endometriosis remains unclear, with multiple theories proposed to explain its development. These include retrograde menstruation with implantation, coelomic metaplasia, direct transplantation, aerosolization, hematogenous or lymphatic spread, and immune system dysfunction. Additional mechanisms such as dissemination through surgical intervention and the metaplastic transformation of pluripotent cells have also been suggested [7].

Scar endometriosis is generally considered to result from the direct implantation of endometrial cells into the abdominal fascia or subcutaneous tissue during surgical procedures, with subsequent stimulation by estrogen promoting their growth [8]. Endometriosis at a trocar port site may develop through peritoneal dissemination of endometrial cells facilitated by pneumoperitoneum or through direct contact of excised lesions with the port tract [9]. Another proposed mechanism suggests that cutaneous endometriosis may arise from the transport of endometrial tissue via lymphatic or vascular routes [10].

Frequent risk factors for AWE include elevated body mass index, multiparity, and surgeries involving the uterine cavity. In our case, a mass at the umbilical optical trocar site was excised 17 years after a laparoscopic cholecystectomy, which had been performed one year after the patient’s last cesarean section. The lower abdominal scar from the two prior cesarean deliveries remained unchanged, with no abnormalities detected on clinical examination or imaging studies. However, in the authors’ view, the most plausible explanation for the development of the lesion at the optical trocar site is the sequence of events described – namely, the cesarean sections, followed by laparoscopy performed relatively soon afterward, and the subsequent slow growth of the lesion in the umbilical region. Although the lesion appeared at the trocar site used during the laparoscopic cholecystectomy, a direct causal relationship with the cholecystectomy cannot be established. The patient had undergone two cesarean sections, which are the most consistently documented iatrogenic risk factor for abdominal wall endometriosis [11]. It is therefore more likely that microscopic endometrial tissue was implanted during one of these cesarean deliveries, with later hormonal stimulation contributing to gradual lesion growth. The pneumoperitoneum generated during the cholecystectomy may theoretically have facilitated redistribution of previously implanted cells, but this remains speculative.

The diagnosis of abdominal wall endometriosis is primarily clinical, typically based on the presence of a mass at the site of a surgical scar that becomes painful during menstruation [12]. An abdominal wall mass (reported in 82–96 % of cases) and pain (41–87 %) are the most common symptoms of AWE [13]. Similarly, in our case, the main reasons for the patient seeking medical attention were pain and a palpable mass in the umbilical region. However, cases presenting with a non-cyclical painful mass or with a long delay between surgery and the onset of symptoms should raise suspicion for other possible conditions [14]. AWE should be differentiated from incisional hernia, granulation tissue, hematoma, abscess, sediment, desmoid fibromatosis, lipoma, infection, soft tissue sarcoma, and metastatic malignant tumours [15]. Gupta et al. reported cases of sporadic desmoid-type fibromatosis occurring at the umbilical trocar site eight months after laparoscopic cholecystectomy. Desmoid fibromatosis is a rare, benign soft tissue tumour arising from the musculoaponeurotic structures. Although it has no malignant potential, it can be locally invasive and may mimic AWE [16].

There is currently no gold standard for the preoperative diagnosis of AWE. On ultrasound, which is a simple, safe, and cost-effective modality, AWE nodules typically appear pear-shaped with a solid hypoechoic or cystic pattern. High-resolution ultrasound is generally sufficient to indicate the need for surgical intervention. Additionally, color Doppler can demonstrate peripheral vascularization consistent with features of endometriotic lesions described in the literature [17]. Other imaging modalities, such as magnetic resonance imaging (MRI) and computed tomography (CT), can help assess the extent of lesions. Although radiologic techniques have high sensitivity, they cannot reliably differentiate between various types of abdominal wall masses. CT imaging can aid in diagnosing, excluding, or suggesting the presence of a mass and delineating its size and nature. For extensive lesions, CT or MRI offers more precise evaluation of the involvement of fascia or rectus abdominis muscles and can help determine whether preoperative planning for abdominal wall reconstruction is necessary [15]. MRI, in particular, provides superior contrast resolution compared to CT or ultrasound [15]. Ultrasound-guided fine needle aspiration can further support the diagnosis and help rule out malignancy prior to surgery. In our described case, a biopsy was also performed prior to definitive surgical treatment to exclude malignancy.

Several treatment options for AWE have been proposed, including pharmacological and surgical approaches; however, the definitive and gold-standard treatment is wide surgical excision with negative margins to prevent recurrence [18]. It is widely accepted that surgical removal of a margin of 5–10 mm of healthy tissue around the lesion is necessary to minimize the risk of recurrence [8]. Specifically, for trocar site endometriosis, wide local excision with at least 5–10 mm of normal surrounding tissue is considered the treatment of choice. In line with this, the surgical margins in our case were at least 7 mm, as confirmed by histopathological findings.

Due to the histological characteristics of scar tissue, pharmacological therapy alone is generally insufficient to achieve a cure. Oral contraceptives and progestins may provide partial symptom relief but do not eliminate the lesion [19]. In a study by Alborzi et al. [20], laparoscopic exploration was performed after resection of abdominal wall masses in 30 patients with AWE, revealing that 28 (93.3 %) also had pelvic endometriosis. Therefore, we believe that postoperative medical therapy should be considered following surgical excision of AWE, in our case, the patient was treated postoperatively with dienogest as adjuvant therapy.

Malignant transformation of peritoneal endometriosis is extremely rare. Among reported cases, clear cell carcinoma arising in an abdominal wall incision is the most common histological type, followed by endometrioid carcinoma [21].

## Study limitations and strengths

This manuscript describes an extremely rare case of trocar port site endometriosis following laparoscopic cholecystectomy and two caesarean sections, contributing valuable information to the limited literature on AWE after non-gynecologic laparoscopic procedures. This study is limited by its design as a case report with a narrative review. The literature search, although improved, is not systematic. Quantitative synthesis is constrained by heterogeneity and incomplete follow-up across published reports. Our follow-up period is too short to assess recurrence. Additionally, the etiology cannot be definitively attributed to the laparoscopic cholecystectomy due to the strong alternative explanation of prior cesarean deliveries.

## Conclusions

Given that iatrogenic implantation is considered a key etiological factor in AWE, preventive measures during surgery are crucial. Strategies include removing trocars only after desufflation of the pneumoperitoneum, using specimen retrieval bags, thoroughly irrigating incisions and trocar sites, and rinsing or replacing any instruments or sutures that may have been contaminated. Avoiding laparoscopic procedures during menstruation and minimizing postoperative complications may further reduce the risk of implantation.

Awareness of abdominal wall and port-site endometriosis is essential not only for gynecologists but also for general surgeons, who frequently evaluate postoperative abdominal wall lesions. When assessing a painful mass at a previous incision or trocar site, AWE should be considered in the differential diagnosis. Wide surgical excision with clear margins remains the treatment of choice.

Given the rarity of reported cases and the lack of standardized diagnostic and follow-up protocols, international collaboration and the development of multicenter registries would significantly enhance understanding of disease patterns, risk factors, and recurrence rates.
